# Correction: Pessali-Marques et al. Musculoskeletal Morphology and Joint Flexibility-Associated Functional Characteristics across Three Time Points during the Menstrual Cycle in Female Contemporary Dancers. *J. Funct. Morphol. Kinesiol.* 2024, *9*, 38

**DOI:** 10.3390/jfmk11010038

**Published:** 2026-01-16

**Authors:** Bárbara Pessali-Marques, Adrian M. Burden, Christopher I. Morse, Gladys L. Onambélé-Pearson

**Affiliations:** 1 Department of Sport and Exercise Sciences, Institute of Sport, Faculty of Science and Engineering, Manchester Metropolitan University, Manchester M1 7EL, UKa.burden@mmu.ac.uk (A.M.B.); c.morse@mmu.ac.uk (C.I.M.); 2 Bastidores—Dance, Research and Training, Palhano, Brumadinho 35460000, Brazil

In the original publication [1], there was a mistake in Table 2 as published. Several rows were misaligned: towards the lower part of the table (from the ‘SJ impulse (Ns)’ row to ‘SJ v_take-off_ (m/s)’ row), some values are displayed one row below their intended position. We have also updated the decimals in values to provide greater details (from below the ‘CMJ v_take-off_ (m/s)’ row). The corrected Table 2 appears below. The authors state that the scientific conclusions are unaffected. This correction was approved by the Academic Editor. The original publication has also been updated.

**Table 2:** Descriptive analysis of the dancers across three menstrual cycle phases (average ± standard deviation). Note that there are no statistically significant differences in raw data.

	Follicular		Ovulatory		Luteal	
Age (years)	23.5 ± 2.94					
Height (m)	1.63 ± 0.05					
Body mass (kg)	67.5 ± 16.0		67.6 ± 15.6		67.8 ± 16.0	
Fat%	25.35 ± 4.53		30.3 ± 6.8		30.81 ± 6.03	
Lean%	69.50 ± 6.94		69.7 ± 6.8		69.18 ± 6.03	
Estimated basal metabolic rate (j)	6487.20 ± 636.34		6460.7 ± 588.1		6449.00 ± 696.53	
Body mass index (Kg/m^2^)	25.29 ± 4.62		25.4 ± 4.5		25.35 ± 4.53	
Fasted glucose (mmol/L)	5.80 ± 2.97		6.06 ± 3.61		5.04 ± 1.16	
Oestrogen (pg/mL)	108.7 ± 46.4		199.28 ± 157.4		161.4 ± 177.3	
Progesterone (ng/mL)	8.8 ± 2.6		9.3 ± 3.0		10.5 ± 2.1	
Relaxin (pg/mL)	4.6 ± 1.3		0.7 ± 0.4		4.9 ± 6.6	
Hips circumference (cm)	98.2 ± 10.6		98.8 ± 10.1		100.9 ± 6.1	
Waist circumference (cm)	79.7 ± 16.9		78.8 ± 16.6		77.5 ± 15.3	
	Dominant	Non-dominant	Dominant	Non-dominant	Dominant	Non-dominant
Calf limb circumference (cm)	36.72 ± 4.06	36.86 ± 3.67	37.11 ± 6.40	37.11 ± 6.39	36.22 ± 7.84	36.05 ± 7.84
Thigh circumference (cm)	53.31 ± 3.30	53.37 ± 3.75	51.72 ± 6.40	51.54 ± 6.61	51.31 ± 6.17	51.31 ± 5.96
ROM_Max_ (°)	135.7 ± 11.98	128.6 ± 13.9	135.6 ± 14.4	130.5 ± 12.8	136.9 ± 14.2	132.5 ± 39.7
Torque_Max_ (Nm)	121.3 ± 37.3	134.3 ± 40.0	129.9 ± 40.5	141.7 ± 36.9	134.7 ± 39.9	136.5 ± 39.7
FSS_ROM_ (°)	102.2 ± 15.8	94.9 ± 13.9	99.0 ± 10.5	96.7 ± 9.8	98.9 ± 9.5	91.6 ± 11.1
FSS_torque_ (Nm)	32.0 ± 13.3	49.2 ± 31.5	46.4 ± 21.6	55.3 ± 29.6	46.7 ± 29.4	34.5 ± 25.9
S_MTU_ (Nm/°)	2.9 ± 1.0	3.3 ± 0.9	3.1 ± 1.3	3.4 ± 0.9	3.2 ± 0.8	3.3 ± 1.3
Elastic potential energy (Nm°)	307.3 ± 105.3	302.2 ± 90.6	334.1 ± 122.6	342.6 ± 111.0	325.9 ± 93.7	341.9 ± 154.4
ST muscle length (cm)	39.2 ± 2.6	39.3 ± 2.5	39.3 ± 1.9	39.5 ± 2.0	39.6 ± 1.9	39.3 ± 1.7
ST muscle width (cm)	3.8 ± 0.5	3.7 ± 0.6	3.8 ± 0.8	3.82 ± 0.6	3.9 ± 0.9	4.1 ± 1.1
ST CSA (mm^2^)	5.2 ± 1.4	5.3 ± 1.2	5.6 ± 1.8	5.4 ± 2.0	5.4 ± 1.8	5.2 ± 1.8
ST fat thickness (mm)	1.3 ± 0.6	1.4 ± 0.6	1.4 ± 0.5	1.4 ± 0.5	1.13 ± 0.4	1.2 ± 0.5
ST muscle lean (mm)	5.2 ± 0.6	5.3 ± 0.8	5.4 ± 0.4	5.4 ± 0.3	5.3 ± 0.6	5.2 ± 0.8
CMJ impulse (Ns)	85.7 ± 32.9	64.0 ± 26.3	70.9 ± 23.8	76.9 ± 15.5	73.0 ± 15.4	74.5 ± 22.9
CMJ ground reaction force (N)	349.0 ± 97.3	356.1 ± 71.3	360.3 ± 95.6	350.4 ± 73.8	376.9 ± 95.7	332.5 ± 64.6
CMJ peak_force_ (N)	412.7 ± 107.7	364.0 ± 83.3	404.6 ± 73.3	385.8 ± 72.3	388.5 ± 64.1	402.4 ± 72.0
CMJ v_take-off_ (m/s)	2.10 ± 0.26		2.05 ± 0.25		2.05 ± 0.27	
CMJ height (m)	0.23 ± 0.06		0.22 ± 0.05		0.22 ± 0.06	
SJ impulse (Ns)	122.3 ± 52.3	106.3 ± 36.7	114.8 ± 40.16	122.6 ± 31.9	123.6 ± 68.0	121.1 ± 36.9
SJ ground reaction force (N)	363.1 ± 102.5	343.7 ± 62.5	357.8 ± 94.1	354.0 ± 74.6	372.2 ± 94.8	336.2 ± 65.0
SJ peak_force_ (N)	426.9 ± 123.8	386.6 ± 105.1	429.4 ± 127.6	402.5 ± 96.9	403.5 ± 76.8	393.3 ± 90.8
SJ v_take-off_ (m/s)	1.98 ± 0.26		1.99 ± 0.28		2.07 ± 0.25	
SJ height (m)	0.20 ± 0.05		0.21 ± 0.06		0.22 ± 0.05	

ROM: range of motion, Max: maximal, FSS: first sensation of stretch, S: stiffness, MTU: muscle-tendon unit, ST: Semitendinosus, CMJ: countermovement jump, SJ: squat jump, CSA: cross-sectional area.
